# Extracting Useful Emergency Information from Social Media: A Method Integrating Machine Learning and Rule-Based Classification

**DOI:** 10.3390/ijerph20031862

**Published:** 2023-01-19

**Authors:** Hongzhou Shen, Yue Ju, Zhijing Zhu

**Affiliations:** 1 School of Management, Nanjing University of Posts and Telecommunications, Nanjing 210003, China; 2 Research Center for Information Industry Integration, Innovation and Emergency Management, Nanjing University of Posts and Telecommunications, Nanjing 210003, China; 3 Nottingham University Business School China, University of Nottingham Ningbo China, Ningbo 315100, China

**Keywords:** social media, microblog, emergency information, machine learning, rule-based classification

## Abstract

User-generated contents (UGCs) on social media are a valuable source of emergency information (EI) that can facilitate emergency responses. However, the tremendous amount and heterogeneous quality of social media UGCs make it difficult to extract truly useful EI, especially using pure machine learning methods. Hence, this study proposes a machine learning and rule-based integration method (MRIM) and evaluates its EI classification performance and determinants. Through comparative experiments on microblog data about the “July 20 heavy rainstorm in Zhengzhou” posted on China’s largest social media platform, we find that the MRIM performs better than pure machine learning methods and pure rule-based methods, and that its performance is influenced by microblog characteristics such as the number of words, exact address and contact information, and users’ attention. This study demonstrates the feasibility of integrating machine learning and rule-based methods to mine the text of social media UGCs and provides actionable suggestions for emergency information management practitioners.

## 1. Introduction

Due to the advances in Internet and mobile communication technologies, social media platforms, such as Twitter and Facebook, have become essential communication tools and significant sources of emergency information (EI), which can be used to support emergency responses [1]. During emergencies, such as natural disasters, serious accidents, or terrorist attacks, users of these platforms easily produce various emergency-related information in the form of texts, pictures, and videos, forming user-generated contents (UGCs). Some of these UGCs provide useful information to emergency information management (EIM) professionals for emergency diagnosis and response, including damage severity, rescue needs, or missing persons [2], and can help allocate resources and improve relief efficiency [3]. Therefore, classifying and verifying the UGCs of social media platforms can generate useful EI and enable social media to play a more active role in emergency management [4].

Nevertheless, emergency-related UGCs produced and disseminated on social media platforms have yet to directly assist the on-site rescue activities of emergency management agencies (EMAs) or volunteers [5]. Despite their wide availability, real-time emergency-related UGCs have varying quality and authenticity due to the heterogenous cognition, motivation, communication skills, emotions, etc., of the authors. Social media platforms are incapable of authenticating all information publishers or all of the information published [6,7], making it necessary to develop efficient approaches to extracting reliable and useful EI from social media UGCs after emergency occurrence. The reliable and useful EI allows decision makers to make more rational emergency responses.

Many researchers have tried to mine EI from social media UGCs through machine learning methods [8,9,10]. However, the results end up with unsatisfactory accuracy and reliability, primarily because these methods rely solely on algorithms, ignoring the role of EIM experts’ knowledge or experience [11]. To mitigate this constraint, this study designed and implemented a machine learning and rule-based integration method (MRIM), which synthesizes machine learning and rule-based classification methods. In this study, we try to answer the following questions: (1) Can the integration of machine learning and rule-based classification be more efficient when extracting useful EI from social media? (2) What characteristics of UGCs affect the performance of the integrated method? (3) How can social media UGCs be better utilized in EIM?

This study focuses specifically on the natural disaster scenario. We tested the integrated method with UGCs on China’s largest microblogging platform—Sina Weibo—on the “July 20 heavy rainstorm in Zhengzhou”. Our results show that this integrated method performs better than pure machine learning methods and pure rule-based methods. We further analyzed how its performance is subject to UGC characteristics and why it encounters misclassification.

Our study enriches the EIM literature by showing the significance and feasibility of integrating UGC characteristics into the machine learning classification process through expert-experience-based rules and extracting useful EI from social media UGCs after an emergency occurs. We also shed light on what characteristics of UGCs impact the performance of the integrated classification method, thus enriching scholarly understanding of how text mining technologies can be designed to more effectively extract EI from social media platforms. Practically, we inform some approaches through which EMAs can incorporate social media UGC analysis into formal EIM.

The rest of this paper comprises four sections. First, we review related research on the utilization of social media in emergencies and extracting information from social media through machine learning methods and rule-based classification methods. We then explain the design of MRIM and present comparative experimental results about its performance. We conclude this paper with discussions about our contributions, limitations, and future research.

## 2. Related Work

### 2.1. Utilization of Social Media in Emergencies

Researchers have noticed that UGCs on social media platforms can be effectively used in different EIM scenarios. They have extensively discussed how the public uses social media to disseminate and obtain EI and how EMAs use social media to publish and gather EI.

Social media has become the first channel through which the public disseminates and obtains emergency-related information [12], such as the location and time of the events, emotions or sentiments, and medical/material help [13,14]. Individuals often provide situational information on social media, and all such information can be amplified and affect broader audiences through social media [15]. For instance, when the 2010 Haiti Earthquake damaged the telephone communication network, social media users disseminated mobile phone short message service (SMS) messages to help locate or rescue victims [16]. Research on social media users during the 2018 Hurricane Florence revealed that different demographic groups have response disparities on social media, which enables EMAs to identify the more sensitive groups in a crisis and provide targeted disaster evolution reports and relief resources to the corresponding demographics [17]. Social media is also an important source for the public to obtain official EI from EMAs. After people are informed about flood disaster mitigation via EMAs’ tweets, individuals can receive updates, distribute supplies, inform requests for help and coordination, and criticize the government [18]. In addition, social media allows users to tap into informal public communications during emergencies [19]. Stieglitz et al. [20] analyzed the structure and sentiment of social media communications for inter-subjective sense making in response to an extreme event and revealed a noticeable difference in emotional tweets between different events.

EMAs have achieved remarkable results in using social media to publish and gather EI. They have used social media to issue official announcements and were recognized as being more interactive with the public [21,22]. For instance, during the 2013 Colorado Flood, Twitter was used by government agencies to provide communication channels and useful information about recovery which were difficult to provide via traditional media [23]. EMAs could gather first-hand information related to an emergency from social media, including people’s emotions, messages of support, and political content, which can more intuitively display experiences, views, and opinions to directly inform emergency rescue operations [24]. After the breakout of COVID-19 in Wuhan, content and sentiment analyses were applied to Chinese microblogging UGCs to understand people’s behaviors and emotions in response to the death of Dr. Wenliang Li [25]. Pradeepa et al. [26] believed that EMAs could monitor the public’s attitudes towards emergencies and take actions quickly by collecting and analyzing social media UGCs. All social media platforms now work on smartphones, which helps EMAs gather more information containing geographical location and obtain location-specific details about the disaster severity [27]. One of the more proactive uses of social media is building a team of trusted volunteers to analyze the information on social media and establish communication with the general public after an emergency. Building a team of trusted volunteers through serious games to evaluate the potential of social media information in early warning disaster management can unite digital volunteers to coordinate EIM and monitor social media communication, leading to better decisions and increasing associated confidence levels [28].

Obviously, the adoption of social media in EIM has been accepted by the public and EMAs. EI extracted from the UGCs on social media is of great value to all kinds of work in emergency management. However, because of the large amount of useless and incorrect information in UGCs, it is critical to find a better automatic information extraction method [29].

### 2.2. Extracting Information from Social Media through Machine Learning

Using machine learning technologies to process massive social media data is efficient and cost effective [30,31]. In general, there are three root learning approaches available in machine learning, including supervised learning, unsupervised learning, and reinforcement learning. The method of machine learning is a generalized inductive process, which uses a group of pre-classification examples to establish classification through training. Currently, machine learning technologies are used extensively and successfully in text-based classification and object recognition, including text mining and sentiment analysis [31,32,33]. Among various supervised learning algorithms, the support vector machine (SVM) has attracted extensive attention because of its excellent generalization ability, optimal solution, and recognition ability compared to text classification tasks [34]. The SVM classifies the given data by finding a hyperplane on the data feature set to achieve the best classification results, and such a hyperplane is used to divide these data into groups [35]. Many researchers believe that the SVM algorithm is a suitable method for text mining, since it has achieved substantial improvements and promising performance over various learning tasks [36]. Collier and Doan [37] collected and classified the tweets based on keywords, and the field study indicated that the comprehensive performance of the results produced by the SVM was better than the naive Bayesian algorithm, especially when the number of training examples was small.

Most machine learning methods used for text mining rely on features from the data, namely, variables or predictors [38]. Using machine learning technologies to extract information from social media is efficient, but it needs to extract features from plain text. One of the “features” is a variable or a predictor in the model, which is similar to the independent variable in regression analysis and makes the classification task more robust. The value of the feature is determined by each word or phrase in the text, and there may be a large number of unique words in social media data, which means that a single social media datum may have many characteristics. However, these unclassified, disordered features may not only make the events of preprocessing and training datasets take longer but can also significantly affect the classification performance of machine learning [39,40]. Accordingly, to improve the performance of machine learning classification, some researchers constructed word vectors to extract features of each document from the original data. For instance, Kadhim et al. [41] and Srinivasa et al. [42] used two feature extraction methods, TF-IDF (term frequency/inverse document frequency) and TF-IDF global, to reduce the dimensionality of datasets. Among all feature extraction methods used for text mining, TF-IDF is commonly used to suppress secondary words and highlight important words in the plain text to classify disordered features [43]. An experiment investigating rapid damage assessment using social media UGCs showed that the combination of TF-IDF and SVM could obtain a better classification effect than other algorithms [44]. A case study conducted on Chinese and Arabic natural language inference tasks also confirmed that appropriately leveraging N-gram helps to achieve a good understanding of the input text and robust behavior when performing this task [45]. Rahman et al. [46] combined TF-IDF and N-gram for the text classification task of SVM and achieved a good classification result.

Text mining based on machine learning is often used to extract specifically required information in EIM. For example, Rudra et al. [9] collected near-real-time status updates on social media and used SVM as text mining technology to discover situational information that could help with emergency rescue activities. Text mining in EIM can also focus on other topics, such as damage evaluation reports [8], situational awareness [47], and dissemination of vaccine information [48]. Most such research is conducted to filter information, as there is lots of low-relevance and low-quality content on social media. To further understand the usefulness of social media information, Nguyen et al. [10] verified the feasibility of machine learning technology to detect crisis-related data on social media immediately, and it was also proposed that the scarcity of labeled data delayed the learning process. This area remains an attractive research topic for determining ways to use machine learning technology to filter useless data and obtain higher-quality social media data.

### 2.3. Rule-Based Classification Method

Although machine learning technologies behave robustly during various text mining tasks, their classification results can easily be affected by the selection of training and test datasets because they rely on training datasets [34]. In addition, in emergency management scenarios, the pure machine learning method cannot integrate the experiences of experts into the classification results, which may affect the support provided for emergency decision making [11,49]. It is a good practice to supplement the machine learning method with the rule-based classification method, which can use some rules developed based on expert experience to judge the information. In doing so, expert knowledge can be incorporated into the classification process [38], and such classification results are well suited for the analysis in comparison with established rule-based learning.

In the rule-based classification method, expert knowledge is expressed by a set of rules. It has an IF (Condition) THEN (Behavior) structure: when the conditions of the rule are met, the rule is triggered, and then the behavior is executed. For instance, when the rules developed by experts according to communication characteristics and user characteristics [50] are triggered, such characteristics can be fully considered in a classification procedure. These characteristics are as important as the text content. The communication characteristics of social media are user feedback formed during the communication process of UGCs, including features such as the number of likes, comments, and shares (or retweets). Researchers have found that the communication characteristics of social media impact the effectiveness of content. For example, social media UGCs related to situational awareness, damage, and rescue locations attract more attention [51], which is also directly proportional to the number of information characteristics [15]. Meanwhile, user characteristics of social media are features that can help portray and distinguish users and are crucial factors when it comes to using UGCs, including features such as the number of blogs previously published and the number of followers and fans. Chen and She [6] found that authenticated users are more active and influential than unauthenticated users. From the perspective of user types, UGCs published by individuals generally present first-hand information and personal experiences [52], while UGCs provided by official accounts usually provide comprehensive reports about affected areas and victims [53].

In the field of EIM, the rule-based classification method has been successfully applied to content analysis of social media during flood disasters and has been used to detect flood extent by combining expert data [54,55,56]. However, to our knowledge, this method has not been used to extract useful EI from social media. We expected that integrating this method with a machine learning method would take advantage of the two and improve the effectiveness of text mining. Hence, we conducted this study.

### 2.4. Research Review

Through a review of related work, it is not difficult to find that most of the existing research on extracting information from social media focus on pure machine learning methods. There is a lack of human intelligence and expert knowledge in the process of information mining. In addition, there are few studies that explore how to identify, extract, and analyze the useful EI from social media and then improve relief efficiency during emergencies. Therefore, this study aims to explore the feasibility of integrating a machine learning and rule-based classification method for extraction of useful EI from social media after an emergency occurs. It also aims to provide suggestions for EMAs to make more effective use of UGCs on social media, further facilitating the integration of the analysis of UGCs into formal EIM.

## 3. Research Design

### 3.1. Research Object

This study chose the social media platform Sina Weibo (https://weibo.com/) as a research setting for three reasons: (a) Weibo, often referred to as “Chinese Twitter”, has become the largest social media platform in China, accommodating 573 million monthly active users as of December 2021 [57]. (b) Weibo allows researchers to write queries and download microblogs under specific topics. (c) Each Weibo microblog (a single post) contains sufficient data, including content, attention, and user attributes. Content is the main body of a microblog and is usually presented as text, pictures, or videos. Attention reflects the public’s attitude towards a microblog, including the number of likes, comments, and shares. User attributes consist of the number of blogs published by the account, and the number of fans and followers of the account.

The emergency in this study, the “July 20 Heavy rainstorm in Zhengzhou”, was a rainstorm that occurred in Zhengzhou, Henan Province, China, from 17 July to 22 July 2021. This natural disaster was caused by extreme rainstorms that led to severe urban waterlogging, river flooding, and landslides. The disaster resulted in 380 people being reported dead or missing in Zhengzhou and caused direct economic losses of CNY 40.9 billion [58]. As shown in Table 1, this emergency attracted the attention of EMAs (such as Zhengzhou Headquarters for Flood Control and Drought Relief) and the general public. Individuals shared extensive near-real-time information through Weibo, forming many Weibo topics directly related to the rainstorm. Among these topics, “# Henan rainstorm mutual assistance #” was launched by the official accounts of the Henan Broadcasting System on 20 July 2021, calling on the public to carry out mutual assistance during this rainstorm. However, although there were over 100,000 microblogs on this topic, many of these microblogs were not clearly expressed and lacked crucial information that could provide direct help in an emergency rescue situation. Therefore, this study chose the original microblogs on the topic of “# Henan rainstorm mutual assistance #” on the Weibo platform and tried to integrate machine learning and rule-based classification methods to extract useful EI from the massive UGCs quickly and effectively.

### 3.2. Research Methodology

**Figure 1:**
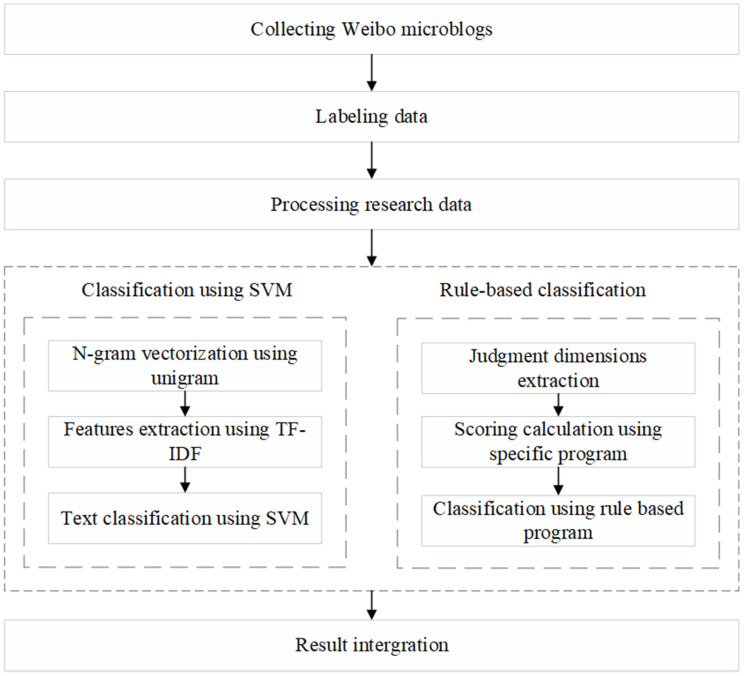
The integration process of MRIM.

The primary purpose of this study was to design and implement an integrated method that can extract useful EI from UGCs on social media to directly support rescue activities during emergencies. Based on a comprehensive analysis of the Weibo microblogs, we decided to integrate machine learning and rule-based classification methods to automatically divide the data into two categories according to whether they can be used to support emergency rescue actions. We named this integrated method MRIM. The integration process of MRIM is shown in Figure 1.

#### 3.2.1. Machine Learning Method

As for the algorithm of machine learning, we chose SVM for the classification based on the following reasons: (a) Compared with other classification algorithms, SVM is better at classifying short text data such as microblogs [59]. (b) SVM can behave robustly in the case of a few training datasets [37]. To verify the applicability, SVM was compared with other machine learning classification algorithms in the experimental evaluation. This study used TF-IDF to extract key phrases, and unigram was applied as N-gram vectorization models with a TF-IDF features extraction method. Such an improvement of TF-IDF could produce a composite weight for each term in a document to suppress secondary words and highlight capital words [60]. The calculation formulas of TF-IDF are defined as follows:

(1) tfi,j=ni,j∑knk,j

(2) idfi=log10|D|(1+|{j:ti∈dj}|) 

(3) $$tfidf_{i,j}=tf_{i,j}\times idf_{i}$$

where *n_i,j_* is the frequency (i.e., the number of times) a word appears in the file *d_j_,* and the denominator is the sum of frequencies of all words appearing in the file *d_j_*; |*D*| is the total number of documents in the corpus indicating the number of files containing the word *t_i_* (the number of files containing *n_i, j_* ≠ 0).

Feature extraction and the SVM algorithm were combined to solve the problem of sparse Weibo microblog data and classify disordered feature sets. These advantages are very meaningful for the research object of this study.

#### 3.2.2. Rule-Based Classification

Based on machine learning classification, this study integrated a rule-based classification method that scores each Weibo microblog through rules developed based on expert experience to judge the usefulness of the microblog in emergency rescue, and such rules are well suited to analysis in comparison with established rule-based learning. As shown in Table 2, the rules in this study need to extract six judgment dimensions from three aspects of microblogs: content characteristics, communication characteristics, and user characteristics.

We invited three experts familiar with social media and disaster management (two researchers from universities and one staff member from the EMAs of local government) to determine the scoring rules of the usefulness of Weibo microblogs using the Delphi method [61]. The experts scored the importance of the above six judgment dimensions at five levels to determine their weights (shown in Table 2), considering the corresponding experts’ familiarity with this dimension.

The implementation procedure of the Delphi method was as follows: First, the first initial questionnaire was designed by us to determine the three experts’ anonymous views on and familiarity with the measures (aspects, dimensions, and parameters) of useful EI on social media, including the importance levels (from the least important (1) to the most important (5)) of each characteristic. Secondly, the second questionnaire was given to all experts (listing the importance levels provided by the other experts), and they were asked to evaluate the others’ opinions according to the results of the others’ first questionnaire. Thirdly, the third questionnaire was used to update the importance level of each measure based on other experts’ opinions, including the evaluation results, average evaluation, and consensus provided by the second questionnaire. Finally, we summarized the results, including all the evaluations, consensus, and remaining issues, and the weight was finally determined by considering the familiarity and the importance levels of each measure provided by each expert.

In the third round of the Delphi method, as the qualified score obtained according to the proportion of useful data in the training dataset was 40.5, and considering the scoring rules, the platform of Weibo, and expert knowledge, the experts also recommended that there is an appropriate score between 35 and 45 which can help us distinguish whether a microblog is useful or not. Accordingly, we selected different integer values between 35 and 45, tested them on some sample data, and found that the classification results were the best when 40 was chosen as the boundary score of the usefulness of microblogs. Finally, for each microblog, the following steps were used to judge its usefulness:

(a) Extract the relevant data of the parameters corresponding to the six judgment dimensions of each Weibo microblog, as shown in Table 2;
(b) Score the six judgment dimensions of each microblog according to the values of parameters;
(c) Calculate the usefulness score of each microblog and classify it as useful or useless.

**Figure 2:**
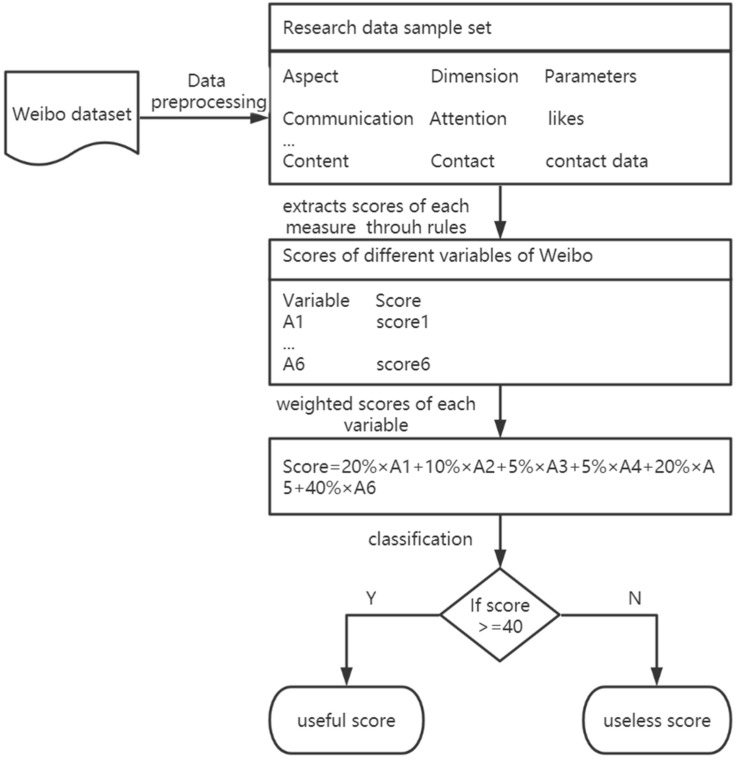
The flowchart of the rule-based classification program.

The above steps (shown in Figure 2) are performed through a Python program.

#### 3.2.3. Classification Result Integration

This study integrated the classification results of machine learning and the rule-based method to form an integrated classification result. Based on the principle of not missing any useful EI as far as possible, any microblog that could support the emergency rescue should be classified as useful EI so as to reduce potential losses during emergencies. Consequently, a microblog will be classified as useless EI only when both of the above two methods classify it as useless, which can be regarded as the integration strategy of MRIM.

### 3.3. Research Data

Our raw data are original microblogs under the topic of “# Henan rainstorm mutual assistance #” on the Weibo platform. We processed them as described below to evaluate MRIM performance.

#### 3.3.1. Collecting Weibo Microblogs

We used a Python crawler to collect Weibo microblogs under the topic of “# Henan rainstorm mutual assistance #” posted from 20 July to 14 August 2021. The starting time was when the “# Henan rainstorm mutual assistance #” topic first emerged, and the ending time was when the Henan Provincial EMA announced that the flood emergency responses were accomplished. For each Weibo microblog, we examined data including text content and the number of likes, shares, comments, microblogs published by each user, fans, and followers on the user’s home page. We then eliminated duplicated microblogs from the collected data and those posted after 4 August 2021 due to their low correlation with emergency rescue. Accordingly, 7979 microblogs posted from 20 July to 3 August 2021 were included in this study.

#### 3.3.2. Labeling Data

To apply machine learning and rule-based classification methods, we need to label the research data manually to determine whether the content of a microblog is useful for emergency rescue based on human judgment. Eight students from our university were recruited as participants to manually label the data in the following steps:

(a) Training participants: We educated participants about the purpose and requirements of the labeling task, and then conducted a pilot test on their labeling performance to ensure everyone was qualified for the task.
(b) Labeling data: We divided 7979 microblogs into four groups and assigned two trained participants to simultaneously label each group. Therefore, each microblog had two results given by different participants. When labeling, participants were required to carefully read and fully understand the content of a microblog and then judge whether the microblog was useful for emergency rescue. The labeled result of each microblog was assigned one of three categories: useful, useless, and uncertain.
(c) Confirming the labeled results: By comparing the two labeled results of each microblog, we could confirm the final labeled result. The final result should be useful or useless, and the confirmation process was as follows:
  (1) If the two labeled results were the same (useful or useless simultaneously), they would be adopted directly.
  (2) If there were two different labeled results for a microblog, or at least one "uncertain" result, the two participants responsible for the labeling would analyze and discuss the results with a third-party researcher to determine the final labeled result.

After manually labeling the research data, 1936 of the 7979 microblogs were labeled useful (marked as 1) and 6043 as useless (marked as 0).

#### 3.3.3. Processing Research Data

We processed the labeled data with Python programs to build a dataset for further text mining. For each Weibo microblog, the data processing included counting the number of words, calculating the sentiment value, and extracting the exact address and contact information.

To count the number of words in a microblog, we removed the HTML elements from the text through regular expression and then segmented the plain text into a set of words using a Chinese text segmentation tool named “Jieba” [62]. Next, we removed the stop words and counted the number of words in the remaining text.

To calculate the sentiment value of a microblog, we traversed the word list after segmentation; checked degree adverbs, negative words, and emotional words; and recorded their positions. We labeled positive and negative emotional words as 1 and −1, respectively, determined the weight of degree adverbs and negative adverbs, and then weighed them with emotional words to calculate the sentiment value. The calculation program builds on multiple specialty dictionaries, including How Net emotion lexicon [63], the National Taiwan University Sentiment Dictionary (NTUSD), the Chinese Dictionary of emotional polarity, and the Chinese emotional vocabulary ontology library of Dalian University of Technology [60]. Sentiment values greater than 0, less than 0, and equal to 0, respectively, represent the positive tendency, negative tendency, and neutral tendency of a microblog.

Next, we extracted the exact address and contact information in two steps. First, we extracted addresses in microblogs with a combinational method of named entity recognition and feature words. Feature words are prevalent in Chinese addresses, reflecting real semantics and address levels [64,65]. Therefore, this study builds on research about address elements [64] to identify and extract address feature words in microblogs through Python programs. As shown in Table 3, the addresses containing 1–5 levels of feature words were defined as exact addresses. Microblogs were given a score of 1 or 0 to represent whether they contained exact addresses. Second, we extracted contact information from microblogs by using a Python program with regular expression. Microblogs were also given a score of 1 or 0 to indicate whether they contained contact information.

After labeling and processing, we obtained scores of three dimensions of a microblog’s usefulness. As extreme values of the three dimensions may distort the overall usefulness score, we set a maximum score for each parameter of the three dimensions to avoid the interference of extreme values in the rule-based classification results, which should exceed 90% of all values of the parameters concerned. We then determined the scoring formula of each parameter according to the non-zero average value of each dimension to facilitate calculation of the scores of parameters. The microblog usefulness dimensions and their parameter settings are shown in Table 4.

After finishing the data processing work, the final research dataset used for text mining was ready.

## 4. Evaluation

### 4.1. Evaluation of MRIM

#### 4.1.1. Overall Performance

To evaluate the overall performance of MRIM in extracting useful and reliable EI from Weibo microblogs, we performed several comparative experiments on MRIM, pure machine learning, and rule-based methods. For machine learning methods, we used the linear kernel in the SVM algorithm, and we used tenfold cross-validation to test the accuracy of the algorithm. To achieve better generalizability, the SVM algorithm used words with a frequency of three or more and unigram as N-gram vectorization models using the TF-IDF features extraction method.

In the comparative experiments, the method using machine learning alone was named Model 1, the method using rule-based classification alone was called Model 2, and MRIM was named Model 3. The common indicators of classification performance included precision (proportion of correct positive prediction to all positive predictions), recall (proportion of correctly predicted positive to all actually positive), and F-measure. As F-measure is the value that can balance the precision and recall, it was used as the primary comparison measurement.

As shown in Table 5, MRIM (Model 3, F-measure = 0.831) proposed in this study performed better than the SVM alone (Model 1, F-measure = 0.714) and rule-based alone methods (Model 2, F-measure = 0.750). The recall rates of Model 1 and Model 2 were comparably low, indicating that these two methods could not effectively detect the useful EI from microblogs to support emergency rescue. In contrast, MRIM achieved the best result in terms of F-measure (0.831) and recall rate (0.777). The improvement in the recall rate could better reflect the prospect of MRIM in EIM, as a high recall rate showed that more useful EI could be detected while ensuring the classification accuracy.

#### 4.1.2. Integration Strategy

According to the integration strategy of MRIM, a microblog will be classified as useful EI when any machine learning and rule-based classification method determines that the microblog is useful. Is there any better way to integrate the classification results? To answer this question, this study attempted to use the score weighting approaches to integrate the scores generated by the two methods.

The score of the machine learning method was based on its classification results. If a microblog was classified as useful, the score would be 100; otherwise, the score would be 0. The score was subsequently combined with the score from the rule-based method to calculate the weighted integrated score of each microblog. If the Integrated_Score was greater than or equal to 40, it would be classified as useful EI. The formula was defined as follows:

(4) Integrated_Score=(1−w)×SVM_Scroe+w×Rule_Based_Score 

**Figure 3:**
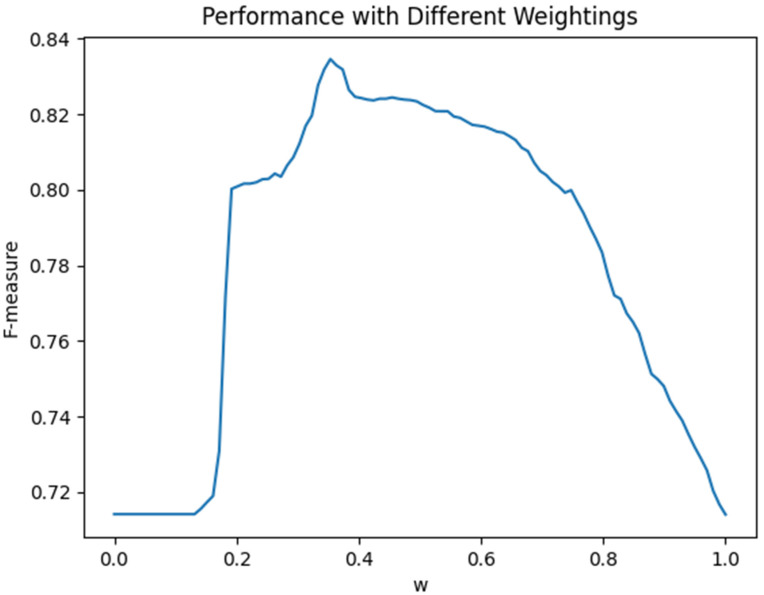
Classification integration performance with different weightings.

In the comparison experiments, the value of *w* was adjusted from 0 to 1, and the change in the F-measure was recorded (Figure 3). Figure 3 illustrates that the performance of the integrated method was higher than the *SVM* alone method (*w* = 0) or the rule-based alone method (*w* = 1). When the value of *w* was set to 0.51, the value of the F-measure reached the peak, which was 0.823 but still lower than MRIM in this study (0.831). This confirms that MRIM has a better integration strategy, which can help us extract as much useful EI from microblogs as possible (with a higher recall rate), and that it behaves robustly during the classification task (with a higher F-measure).

#### 4.1.3. Machine Learning Algorithm

As mentioned above, the SVM algorithm was used in MRIM because it had achieved good results over various text classification tasks [34], especially when the processing object was short text from social media. One of the main reasons for integrating SVM and rule-based methods is that these two methods complement each other [38]. The SVM algorithm provides a good classification performance for text mining without considering word order, while the rule-based method integrates human judgment into the classification process by using the rules developed based on expert experience.

To verify whether SVM was indeed the most suitable machine learning algorithm for our dataset, this study compared it with two other popular machine learning algorithms: the naive Bayesian (NB) and decision tree (DT) algorithms. These were chosen because they are suitable for our smaller dataset and further integration. We compared each algorithm and their combinations. In the comparative experiments, we used the integration strategy of MRIM; that is, if any algorithm classified a microblog as useful EI, the integration method would classify the microblog as such. Table 6 lists the results.

As shown above, the performance of the NB alone method (Model 4, F-measure = 0.656) and the DT alone method (Model 5, F-measure = 0.709) was not as good as that of the SVM alone method (Model 1, F-measure = 0.714), which confirms the superiority of using the SVM algorithm in MRIM. Table 6 also shows that MRIM (Model 3, F-measure = 0.831) has better performance than the integration methods of NB+SVM (Model 6, F-measure = 0.675) and DT+SVM (Model 7, F-measure = 0.764), indicating that the expert experience provided by the rule-based method is helpful for improving the classification results.

### 4.2. Influence of Microblog Characteristics on Classification Results

We performed a follow-up study to explore the association between MRIM performance and microblog characteristics. We investigated three aspects of each microblog, including the number of words, the exact address and contact information, and the attention it received.

The number of words in text is important for text mining. It is thus worthwhile to clarify how the number of words affects the performance of MRIM. For this purpose, all microblogs (in total 7979) were divided into ten groups based on the number of words they contained. The first group contained 797 microblogs with the least number of words, the second group focused on 798 microblogs with the second least number of words, and the last group also contained 798 microblogs but with the largest number of words. The SVM alone method and MRIM were applied to each group, and the F-measure values were recorded. The results are shown in Table 7.

The data in Table 7 indicate that the more words within a microblog, the more robust the classification performance of both the SVM alone method and MRIM (with F-measure values higher than 0.5). These results imply that microblogs with a small number of words cannot provide enough information. Neither the SVM alone method nor MRIM can complete the information extraction work to a satisfactory level.

**Figure 4:**
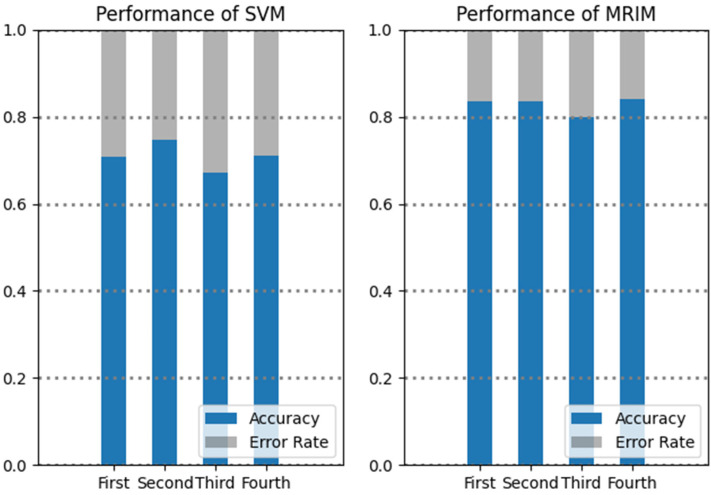
The performance on groups with different amounts of address and contact information.

During emergencies, the exact address and contact information can play a crucial role in rescue activities, so they are one of the focuses in EIM. To investigate the impact of exact address and contact information on classification results, we divided all microblogs into four groups according to whether they contained an exact address or contact information. The first group comprised 5461 microblogs containing both an exact address and contact information. The second group comprised 1202 microblogs containing contact information only, and the third comprised 331 microblogs containing exact address information only. The fourth group comprised 985 microblogs containing neither exact address information nor contact information. Again, the SVM alone method and MRIM were applied to each group, and the F-measure values were recorded. Figure 4 shows the performance of the two methods used on the four groups. We found that MRIM overall performed better than the SVM alone method. However, it is worth noting that both methods had unsatisfactory performance in the third group. A further analysis of the original microblogs of the third group showed that almost 40% of these microblogs had the problem of non-standard writing of exact addresses (abbreviations, regional habits, etc.), and the pattern of word usage was chaotic. Consequently, the keyword-dependent SVM algorithm failed to effectively detect useful EI from microblogs, which also affected the performance of MRIM.

**Figure 5:**
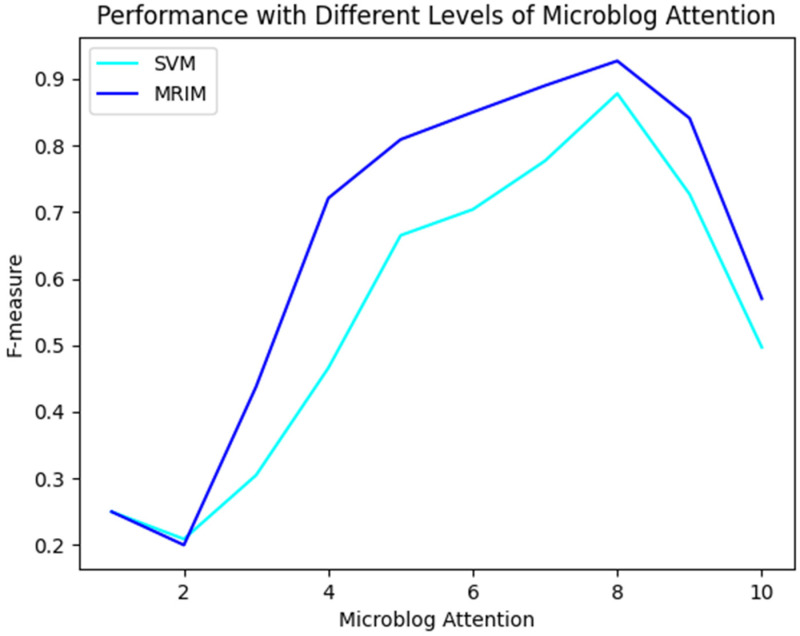
The performance on groups with different levels of microblog attention.

As discussed earlier, the communication characteristics of social media can reflect the effectiveness of UGCs’ content to a certain extent. Therefore, we conducted experiments to study the influence of microblog attention on classification performance. We divided microblogs into ten groups based on the attention they received (measured by the sum of the numbers of likes, comments, and shares). The first group contained 797 microblogs with the smallest amount of attention, the second group focused on 798 microblogs with the second smallest amount of attention, and the last group also contained 798 microblogs but with the largest amount of attention. Both the SVM alone method and MRIM were applied to each group. The variation curves of the F-measure values are shown in Figure 5, indicating that MRIM performs better than the SVM alone for almost all groups, especially the third to sixth groups. According to previous research and preliminary observation, microblogs that receive more attention are often of higher quality and provide more rational textual expression. All such microblogs should be more accurately detected by the SVM alone method and MRIM. Surprisingly, neither the SVM alone method nor MRIM performed well in the groups that received too much attention, such as the ninth and tenth groups. Following further analysis, we found that about 50% of these microblogs were reports of emergency situations generated by professional media. These microblogs tend to be comprehensive in content and receive a lot of attention. Although they have sufficient information features, they are not able to directly contribute to emergency rescue activities [53]. Therefore, we should pay more attention to the microblogs posted by individuals and private organizations, especially those generated by victims and bystanders at the scene of an emergency.

### 4.3. Misclassification Analysis

To improve the classification results of MRIM and lay a foundation for better integration methods, we also identified the reasons for misclassification by MRIM.

We began by performing a detailed analysis of the microblogs that were useless but were mistaken as useful EI. One important finding was that some of these microblogs simply copied the content generated by professional media, which generally provided comprehensive reports on disaster severity and victims. While they could not support emergency rescue actions, their adequate expression and high levels of attention render them easily classified as useful EI. Another important finding was that the classification result of the SVM algorithm was sensitive to missing feature data. The SVM alone method has no strategy to deal with missing values when there are few information features or incomplete vector data in microblogs. Meanwhile, SVM requires the samples to be linearly separable in the feature space, so the quality of the feature space is critical to the performance of SVM. Missing feature data will affect the quality of training results, and all such microblogs may be classified as useful EI by machine learning technology.

We then conducted in-depth analysis of the microblogs that were useful but mistaken as useless EI. There were multiple reasons for their misclassification. First, these microblogs contained informal and colloquial Chinese expressions, which prevented the SVM alone method and MRIM from detecting key information features. Second, the exact addresses and contact information in some useful microblogs were expressed irregularly or even incorrectly, resulting in these valuable information features being incorrectly identified. Finally, some useful microblogs contained fewer words, so their information features were difficult to detect using the classification methods. However, integrating the SVM algorithm and the rule-based method can complement each other, reducing classification errors.

## 5. Discussion and Conclusions

Social media UGCs produced during emergencies are regarded as informative for supporting emergency responses. However, researchers inadequately explored how useful EI can be efficiently extracted from these UGCs and incorporated into the decision-making process of emergency management. To fill this knowledge gap, this study proposes an extracting method (named MRIM) that integrates a machine learning method (SVM algorithm) and a rule-based classification method. Testing the MRIM on microblog data about the “July 20 heavy rainstorm in Zhengzhou” posted on China’s largest social media platform (Sina Weibo), we found that the MRIM has better EI extraction performance than machine learning alone and rule-based alone methods, and that its performance is associated with specific characteristics of UGCs, including the number of words, the exact address and contact information, and the amount of attention received.

### 5.1. The Incorporation of Expert Experience Increases the Effectiveness of the Integrated Method

This study adds to the EIM literature by developing a new method integrating machine learning and rule-based classification methods to effectively extract useful EI from social media UGCs. The method, MRIM, builds on prior studies emphasizing the significance of expert experience [11,49] and UGC characteristics beyond textual content (e.g., communication characteristics and user characteristics; see [12,15,50,52]) in the mining of UGCs. It incorporates various UGC characteristics into the classification process through rules developed on the basis of expert experience, significantly outperforming machine learning alone or rule-based alone methods in identifying useful EI from massive UGCs. In terms of the integration strategy of machine learning and the rule-based classification method, we take a more cautious approach: a microblog will be classified as useful EI when any one of the two methods determines that the microblog is useful. This is because rescue efforts in emergencies are so important that we would rather mistakenly extract useless EI than miss out on truly useful EI. In contrast, SVM and other traditional word-based machine learning algorithms underperform because they rely heavily on keywords [11,34]. They perform better after integrating rule-based classification methods, suggesting the feasibility of integrating expert experience through rule-based methods into machine learning methods to extract useful EI from social media UGCs.

### 5.2. The Number of Words, Exact Address and Contact Information, and Users’ Attention Affect the Performance

By identifying which UGC characteristics can heighten classification performance, this study also enriches scholarly understanding of how text mining technologies can be designed to extract EI more effectively. Comparative experiments on the number of words reveal that UGCs with fewer information features significantly degrade the SVM algorithm’s classification performance, confirming that the SVM method is sensitive to missing data in classification tasks [34]. For particularly short microblogs, the SVM’s reliance on keywords’ co-occurrence is a shortcoming, and other classification methods are required to introduce more clues [38]. Therefore, researchers need to pay special attention to the use conditions of different classification methods to ensure the classification performance through the complementarity of methods.

While exact address and contact information is often used to seek help or rescue [12,27], our results show that the casual writing of exact addresses in UGCs inhibits the performance of machine learning algorithms during classification tasks. Some UGCs have confusing word patterns or text formats, reducing their chances of being recognized by other users [51], which also prevents our Python programs from identifying addresses and contact information more accurately. Therefore, both MRIM and the SVM alone are unable to effectively detect useful EI from such social media UGCs. This also emphasizes that the key role of address and contact information in emergency response cannot be ignored.

Finally, we found that the SVM algorithms misclassified many UGCs that received a high amount of attention, about 50% of which were comprehensive reports about affected areas and victims generated by official media agencies. This finding is consistent with Kim and Park’s view that official media generally report events more comprehensively but cannot provide instant help in EM [53]. The ultimate goal of this study is to determine EI that supports emergency rescue activities; therefore, the role of comprehensive reports by official media agencies is minimal and causes disruption.

### 5.3. The Practical Implications for Better Use of Social Media UGCs in EIM

This study informs EIM practice by showing the promising prospect of using MRIM to efficiently extract EI from the massive number of social media UGCs. Though perceived as important EI sources by EMAs and the public, social media UGCs have not been included in the formal emergency management decision-making procedures due to the daunting data volume and heterogeneous content quality [5]. Our study suggests that EMAs exploit EI-related UGCs by developing EI mining mechanisms with social media platforms or technology companies. It is also worthwhile for EMAs to stay alert to individual and private organizational users of social media during EIM, paying particular attention to the UGCs generated by individuals at the site of an emergency. They may encourage social media users to post more comprehensive and accurate information, such as precise addresses and contact information. Additionally, social media platforms can implement targeted functions for emergency rescue (e.g., developing templates), guiding users to provide more detailed and accurate information during emergencies.

### 5.4. Limitations

Our study has three limitations. First, our proposed integrated classification method proved effective in the rainstorm emergency scenario, indicating that this integration idea is generalizable, but its rules are not directly applicable to other types of emergencies. Different emergencies will require different expert experience and judgment rules, requiring integration methods to be customized and tested across scenarios. Second, the integrated method is designed and implemented based on the Weibo platform and thus cannot be directly applied to other social media platforms. However, the main idea of integrating machine learning and rule-based classification can be replicated on similar social media platforms, such as Twitter. Last, the use of the Delphi method in this study was relatively simple and only three experts were invited. We believe it is still necessary to invite more experts to increase the authority of rule-based methods.

### 5.5. Future Research

Scholars can build on this study to develop new and effective integrated classification methods. The starting point could be using larger datasets in other types of emergencies to verify and strengthen the effectiveness and generalizability of our proposed method. Moreover, researchers can test more characteristics of social media UGCs to explore new approaches that can efficiently and reliably extract EI from UGCs. For instance, they could include temporal and spatial features of UGCs, visual and sound formats (e.g., pictures, videos, emojis), the behavior of users, etc., in classification methods. Finally, given the importance of address information in UGCs for classification performance, it is worthwhile to build a professional address dictionary and develop a more intelligent method to improve address extraction performance.

## Figures and Tables

**Table 1:** Five important fragments of the “July 20 Heavy rainstorm in Zhengzhou”.

Date	Group	What to Do
7.19	EMAs	Issued a red rainstorm warning signal
7.20	EMAs	Issued at least three red rainstorm warning signals
7.20	General Public	“Henan heavy rain”, “Zhengzhou Metro Line 4 has become a water curtain cave”, “Henan rainstorm mutual assistance”, and other topics were posted as Weibo hot search topics
7.21	EMAs	Decided to upgrade the flood emergency response level from II to I
7.23	EMAs	Decided to reduce the flood emergency response level from III to IV

**Table 2:** Dimensions of judgment rules for microblog usefulness.

Aspects	Dimension Names	Parameters	Variable	Weights
Communication characteristics	Attention score	Number of likes	A1	20%
		Number of comments		
		Number of shares		
User characteristics	User score	Number of blogs previously published	A2	10%
		Number of followers		
		Number of fans		
Content characteristics	Emotional score	Sentiment value	A3	5%
	Text word score	Number of text words	A4	5%
	Exact address score	Whether it has an exact address	A5	20%
	Contact score	Whether it has contact information	A6	40%

**Table 3:** Exact address feature words in Weibo microblogs.

No.	Address Level	Feature Words
1	Township street level	towns (‘镇’), townships (‘乡’), offices (‘办事处’), neighborhood committees (‘居委会’), communities (‘社区’), streets (‘街道’), etc.
2	Road village level	village (‘村’), group (‘组’), team (‘队’), Li (‘里’), yuan (‘园’), Zhuang (‘庄’), Nong (‘弄’), Tou (‘头’), Qiao (‘桥’), Kou (‘口’), Tian (‘田’), Dian (‘店’), island (‘岛’), road (‘路’), street (‘街’), Hutong (‘胡同’), etc.
3	Point of interest	hospitals (‘医院’), buildings (‘大厦’), squares (‘广场’), restaurants (‘饭店’), centers (‘中心’), buildings (‘大楼’), fields (‘场’), halls (‘馆’), hotels (‘酒店’), hotels (‘宾馆’), markets (‘市场’), gardens (‘花园’), guest houses (‘招待所’), centers (‘中心’), universities (‘大学’), factories (‘厂’), bureaus (‘局’), dormitories (‘宿舍’), etc.
4	Street level	slope (‘坡’), lane (‘巷’), alley (‘胡同’), village committee (‘村委会’), house (‘号’), residence (‘馆’), garden (‘居’), community (‘小区’), apartment (‘公寓’), courtyard (‘号院’), garden (‘花园’), building (‘大厦’), square (‘广场’), etc.
5	Detailed address level	unit (‘单元’), floor (‘层’), room (‘室’), building (‘栋’), building (‘号楼’), block (‘幢’) building (‘座’), etc.

**Table 4:** Microblog usefulness dimensions and their parameter settings.

Category	Corresponding Parameters	Non-Zero Average	Scoring Formula	Maximum Score
Weibo attention score	Number of likes	196.27	Number of likes/10	33.3
	Number of comments		Number of comments/10	33.3
	Number of retweets		Number of retweeted/10	33.3
Weibo user score	Number of blogs previously published	2299.05	Number of blogs previously published/100	33.3
	Number of concerns		Number of concerns/100	33.3
	Number of fans		number of fans/100	33.3
Weibo content score	Emotional value	5.97	Positive emotional value	100
			Negative emotional value	100
	Number of text words	35.91	Number of text words	100
	Whether it has contact information	1	Existence is 100; non-existence is 0	100 or 0
	Whether it has an exact address	1	Existence is 100; non-existence is 0	100 or 0

**Table 5:** Classification performance comparison.

Model	Precision	Recall	F-Measure
Model 1 SVM	0.916	0.585	0.714
Model 2 Rule based	0.919	0.633	0.750
Model 3 MRIM	0.894	0.777	0.831

**Table 6:** Performance of different algorithm combinations.

Model	Method	Precision	Recall	F-Measure
Model 4	NB	0.934	0.506	0.656
Model 5	DT	0.817	0.626	0.709
Model 1	SVM	0.916	0.585	0.714
Model 6	NB+SVM	0.914	0.535	0.675
Model 7	DT+SVM	0.809	0.724	0.764
Model 3	Rule based+SVM	0.894	0.777	0.831

**Table 7:** Classification results of datasets grouped by the number of words.

Group No.	Average Number of Words	F-Measure (SVM)	F-Measure (MRIM)
1	3.16	0.222	0.250
2	8.38	0.274	0.348
3	13.15	0.241	0.379
4	18.18	0.458	0.635
5	23.94	0.525	0.756
6	30.44	0.662	0.792
7	39.27	0.696	0.851
8	51.07	0.757	0.880
9	69.19	0.831	0.925
10	97.73	0.867	0.898

## Data Availability

By examining the event timelines and the associated hashtags on the popular Chinese social media site Sina Weibo (https://weibo.com/login.php), microblogs under “# Henan rainstorm mutual assistance #” were taken as the research data.
